# Carriage of the V279F Null Allele within the Gene Encoding Lp-PLA_2_ Is Protective from Coronary Artery Disease in South Korean Males

**DOI:** 10.1371/journal.pone.0018208

**Published:** 2011-04-05

**Authors:** Yangsoo Jang, Dawn Waterworth, Jong-Eun Lee, Kijoung Song, Sujin Kim, Hyo-Soo Kim, Kyung Woo Park, Hyun-Jai Cho, Il-Young Oh, Jeong Euy Park, Bok-Soo Lee, Hyo Jeong Ku, Dong-Jik Shin, Jong Ho Lee, Sun Ha Jee, Bok-Ghee Han, Hye-Yoon Jang, Eun-Young Cho, Patrick Vallance, John Whittaker, Lon Cardon, Vincent Mooser

**Affiliations:** 1 Division of Cardiology, Cardiovascular Genome Center, Yonsei University College of Medicine, Yonsei, South Korea; 2 Genetics Division, Medicine Discovery and Development, Research and Development, GlaxoSmithKline, Philadelphia, Pennsylvania, United States of America and Greenford, United Kingdom; 3 DNA Link Inc. Seoul, South Korea; 4 Department of Internal Medicine and Cardiovascular Center, Seoul National University Hospital, Seoul, South Korea; 5 Division of Cardiovascular Disease, Samsung Medical Center, Sung Kyun Kwan University, Seoul, South Korea; 6 Department of Food and Nutrition, College of Human Ecology, Yonsei University, Seoul, South Korea; 7 Department of Epidemiology and Health Promotion, Institute for Health Promotion, Graduate School of Public Health, Yonsei University, Seoul, South Korea; 8 Center for Genome Science, National Institute of Health, Seoul, South Korea; 9 Medicine Discovery and Development, Research and Development, GlaxoSmithKline Philadelphia, Pennsylvania, United States of America and Greenford, United Kingdom; Innsbruck Medical University, Austria

## Abstract

**Background:**

The Asia-specific *PLA2G7* 994G-T transversion leads to V279F substitution within the lipoprotein-associated phospholipase-A2 (Lp-PLA_2_) and to absence of enzyme activity in plasma. This variant offers a unique natural experiment to assess the role of Lp-PLA_2_ in the pathogenesis of coronary artery disease (CAD) in humans. Given conflicting results from mostly small studies, a large two-stage case-control study was warranted.

**Methodology/Principal Findings:**

*PLA2G7* V279F genotypes were initially compared in 2890 male cases diagnosed with CAD before age 60 with 3128 male controls without CAD at age 50 and above and subsequently in a second independent male dataset of 877 CAD cases and 1230 controls. In the first dataset, the prevalence of the 279F null allele was 11.5% in cases and 12.8% in controls. After adjustment for age, body mass index, diabetes, smoking, glucose and lipid levels, the OR (95% CI) for CAD for this allele was 0.80 (0.66–0.97, p = 0.02). The results were very similar in the second dataset, despite lower power, with an allele frequency of 11.2% in cases and 12.5% in controls, leading to a combined OR of 0.80 (0.69–0.92), p = 0.002. The magnitude and direction of this genetic effect were fully consistent with large epidemiological studies on plasma Lp-PLA_2_ activity and CAD risk.

**Conclusions:**

Natural deficiency in Lp-PLA_2_ activity due to carriage of *PLA2G7* 279F allele protects from CAD in Korean men. These results provide evidence for a causal relationship between Lp-PLA_2_ and CAD, and support pharmacological inhibition of this enzyme as an innovative way to prevent CAD.

## Introduction

Elevated levels of lipoprotein-associated phospholipase A_2_ (Lp-PLA_2_), an enzyme involved in the generation of pro-inflammatory products in atherosclerotic plaques, have been consistently associated with an increased risk for coronary artery disease (CAD) and other vascular diseases in Caucasian and other ethnic groups, including Koreans. In a recent meta-analysis incorporating 79036 participants from 32 prospective studies the risk of developing cardiovascular diseases was increased by 11% for each standard deviation (SD) unit increase in Lp-PLA_2_ activity, after correction for other cardiovascular risk factors [1]. The amplitude of the association with CAD was comparable to that of non-HDL cholesterol or systolic blood pressure in these populations. These epidemiological studies have prompted the discovery of pharmacological agents which would inhibit this enzyme and so may prevent the development of cardiovascular diseases. Such agents have shown promise in pre-clinical and early clinical development [2] and are presently in late-stage development [3], [4].

A study of Lp-PLA_2_ activity in Japanese subjects identified a non-functional (null) V279F allele within the Lp-PLA_2_–encoding gene *PLA2G7*, due to a G to T transversion in exon 9 at position 994 [5]. At least 10 separate reports [5]–[14] have demonstrated that homozygous carriers of this variant are lacking the enzyme in plasma and that heterozygous carriers have around 50% the activity of individuals carrying two copies of the wild-type allele. The 279F null allele is relatively frequent in Japan, with approximately 25% and 2% of the population carrying one or two copies[6], respectively, and its prevalence shows a declining gradient towards the West, with intermediate frequencies in China and Korea[8], rare carriers in the Middle East [15] and almost complete absence in Europeans. This discovery provides a natural experiment to gain insight into the causal contribution of Lp-PLA_2_ to the pathogenesis of CAD, and as such shall complement epidemiological studies and contribute to our understanding of the causality of Lp-PLA_2_ in atherogenesis in humans. Other common variants within *PLA2G7* have been reported, and have minimal, if any effects on Lp-PLA_2_ activity as compared to the V279F. In particular, the most well characterized of those, A379V confers only a 7.2% difference in Lp-PLA_2_ activity between AA and VV homozygotes[16].

Several reports have been published on the association between the V279F allele and CAD in Asians. An early study in Japanese subjects showed an increased risk for myocardial infarction (MI) and stroke in carriers of 279F variant[6], [13], whereas a subsequent study showed only a male- and high cholesterol-specific increased risk [17] . Others reported no association in either Japanese or Chinese subjects[7], [18]; lastly a decreased risk of CAD was observed in male carriers of the 279F null allele from Korea [8]. As small sample size (i.e. <1000 CAD cases) and multiple-testing issues due to subgroup analyses were likely factors in the disparity of the results, an appropriately powered study was warranted to better estimate the effect of the null allele on CAD risk. Here, we report the results from a two-stage case-control analysis we performed in men from South Korea. The first dataset (Study 1) comprised 2890 cases diagnosed with relatively premature CAD and 3128 controls clinically devoid of the disease. As a positive control to test for any bias in ascertainment, we used a common (allele frequency ∼50%) single nucleotide polymorphism (SNP) marker within the 9p21 *CDKN2A/B* locus which has been robustly associated with CAD in European [19]–[21] and in Asian populations [19], [22], including in Korea [23], [24]. Recent meta-analysis of the effect of 9p21 on CAD risk in 22 studies comparing a total of 35877 cases and 95837 controls showed that carriage of 1 copy of the risk allele is associated with a ∼25% increase in the risk of CAD. [25] The *PLA2G7* V279F variant was subsequently tested within a second, independent group of 877 cases and 1230 controls (Study 2).

## Results

A total of 2890 CAD cases and 3128 non-CAD controls were included in Study 1 (Table 1). Given the design of the study, CAD cases were younger than controls. As expected and despite their younger age, cases had a larger BMI and higher levels of glucose and lipids, with lower HDL-cholesterol levels, and a larger proportion of them were smokers or diabetic. The majority of CAD cases were on statins, which may explain the lower levels of total and LDL-cholesterol. After correction for statin usage, LDL-cholesterol levels were slightly higher in cases than controls.

**Table 1:** Characteristics of the participants in Studies 1 and 2.

	Study 1			Study 2		
Characteristic	CAD	Control	*p* value*	CAD	Control	*p* value*
n	2890	3128		877	1230	
Age (yrs)	51.1±6.5	58.4±6.0	3.5E-267	56.6±8.2	52.7±9.0	1.0E-28
BMI (kg/m^2^)	25.3±2.9	24.0±2.8	6.8E-57	25.1±2.9	24.2±2.6	1.8E-11
Glucose (mmol/L)	6.3±2.4	5.1±1.1	2.6E-120	5.69±1.9	5.08±0.67	7.9E-06
Triglycerides (mmol/L)	1.57(0.11–23.5)†	1.53(0.34–8.9)	0.03	1.50(0.36–10.8)	1.33(0.17–8.5)	1.8E-07
Total cholesterol (mmol/L)	4.62±1.1	4.94±0.91	3.9E-35	4.34±1.1	5.07±0.90	8.6E-54
HDL-cholesterol (mmol/L)	1.06±0.26	1.19±0.30	5.9E-63	1.14±0.30	1.27±0.34	7.4E-21
LDL-cholesterol (mmol/L)	2.74±0.99	2.96±0.86	9.2E-23	2.43±0.90	3.09±0.85	1.2E-53
Corrected LDL-cholesterol (mmol/L)**	3.11±1.2	2.96±0.86	0.01	2.75±1.02	3.09±0.85	1.4E-18
Myocardial Infarction (%)	42%	0%	na	46%	0%	na
Diabetes (%): Missing *n*	29%: 208	5%: 85	1.2E-132	29%: 0	0%: 0	1.1E-108
Statin (%): missing *n*	58%: 385	0.1%: 640	1.0E-267	59%: 4	0.1%: 159	1.4E-227
Smoking (%): missing *n*	83%: 337	59%: 73	3.6E-89	85%: 3	74%: 2	3.1E-09

Values are mean ± SD.

† Value is median (minimum - maximum).

**LDL-cholesterol levels were adjusted for the use of statins by multiplying levels by 1.25.

*Continuous data were tested using 2-tailed Student t-test or Wilcoxon test and categorical data were tested using Chi-square test for comparison between CAD and controls.

na: not applicable.

RS10757274 within the 9p21 locus was used as a positive control for genetic association with CAD. As expected, the risk allele (G) frequency was significantly higher among CAD cases, and in the subgroup with MI (Table 2 lower panel). The *PLA2G7* 279F null allele (Table 2 upper panel) was slightly under-represented among cases with CAD (11.5%) and MI (11.2%) compared to controls (12.8%).

**Table 2:** Genotype (n, %) and allele frequencies of V279F (*PLA2G7*) and RS10757274 (9p21) in Studies 1 and 2.

	Study 1					Study 2				
	CAD	MI	Control	p1	p2	CAD	MI	Control	p1	p2
***PLA2G7***										
VV n(%)	2205(76)	876(77)	2345(75)	0.10^a^	0.17^a^	692(79)	317(77.9)	933(76)	0.44^a^	0.23^a^
VF n(%)	564(20)	217(19)	679(22)			174(20)	88(21.6)	264(21)		
FF n(%)	40(1)	16(1)	53(2)			11(1)	2(0.5)	20(2)		
MAF* (%)	11.5	11.2	12.8	0.03^b^	0.10^b^	11.2	11.3	12.5	0.21^b^	0.37^b^
Missing n(%)	81(3)	25(2)	51(1)			0(0)	0(0)	13(1)		
**9p21**										
AA n(%)	693(24)	256(23)	979(31)	1.4E-11^a^	2.4E-09^a^					
AG n(%)	1402(49)	556(49)	1485(47)							
GG n(%)	698(24)	280(25)	586(19)							
MAF* (%)	50.1	51.1	43.6	1.7E-12^b^	3.9E-10^b^					
Missing n(%)	97(3)	42(4)	78(2)							

*Minor Allele Frequency. MI: myocardial infarction.

p1: p value for comparison of CAD *vs* control; p2: p value for comparison of myocardial infarction *vs* control.

a p value for comparison of genotype frequency;

b p value for comparison of allelic frequency.

Logistic regression analysis was performed to adjust for differences in various cardiovascular risk factors between the cases and the controls (Table 3). Smoking, diabetes, BMI, glucose, triglycerides and HDL-cholesterol levels were significantly associated with CAD. After inclusion of these covariates into the model, each copy of the 9p21 locus SNP was associated with an increased risk of CAD of 1.28 (95% CI 1.16–1.42, p = 2.4×10^−6^). Conversely, the *PLA2G7* 279F allele was associated with a reduced CAD risk of 0.80 (0.66–0.97, p = 0.02), and the effect tended to be gene-dosage dependent, with an OR 0.79 (0.64–0.98) and 0.69 (0.33–1.44) for carriers of one and two copies of the 279F allele, respectively. The protective effect of the null allele was slightly more pronounced for CAD cases with MI [OR 0.72 (0.56–0.94), p = 0.01].

**Table 3:** Logistic regression model of CAD and MI in Studies 1 and 2.

	Study 1				Study 2			
	CAD		MI		CAD		MI	
Characteristic	OR (95% CI)	*p* value	OR (95% CI)	*p* value	OR (95% CI)	*p* value	OR (95% CI)	*p* value
Smoking	2.85 (2.31–3.51)	7.8E-23	5.52 (3.93–7.77)	1.1E-22	2.15 (1.68–2.75)	1.3E-09	2.68 (1.89–3.79)	2.6E-08
Diabetes*	2.01 (1.36–2.98)	0.0005	2.00 (1.17–3.42)	0.01				
BMI (per 1 kg/m^2^)	1.06 (1.03–1.10)	0.0005	1.06 (1.01–1.11)	0.01	1.10 (1.06–1.14)	1.9E-07	1.11 (1.06–1.16)	1.2E-05
Glucose (per 1 mmol/L)	1.46 (1.32–1.61)	5.4E-14	1.39 (1.23–1.58)	3.2E-07	1.45 (1.32–1.59)	1.2E-14	1.46 (1.29–1.65)	9.1E-10
Triglycerides (per 1 mmol/L)	0.81 (0.74–0.87)	6.9E-08	0.79 (0.70–0.88)	1.7E-05	1.03 (0.93–1.14)	0.6	1.0 (0.88–1.14)	0.96
HDL-cholesterol (per 1 mmol/L)	0.13 (0.09–0.18)	2.4E-29	0.09 (0.06–0.16)	1.8E-20	0.28 (0.20–0.39)	2.1E-13	0.26 (0.17–0.40)	9.9E-10
*PLA2G7* F vs V	0.80 (0.66–0.97)	0.02^a^	0.72 (0.56–0.94)	0.01^a^	0.80 (0.65–0.98)	0.04^a^	0.77 (0.59–1.01)	0.06^a^
FF vs VV	0.69 (0.33–1.44)	0.07^b^	0.60 (0.22–1.60)	0.05^b^	0.63 (0.28–1.42)	0.12^b^	0.21 (0.05–0.95)	0.08^b^
VF vs VV	0.79 (0.64–0.98)		0.71 (0.53–0.95)		0.81 (0.64–1.02)		0.86 (0.64–1.15)	
9p21 G vs A	1.28 (1.16–1.42)	2.4E-06	1.44 (1.22–1.71)	2.5E-05				
GG vs AA	1.64 (1.34–2.01)	3.6E-06	2.07 (1.47–2.91)	1.0E-04				
GA vs AA	1.14 (0.96–1.35)		1.60 (1.20–2.14)					

	Meta							
	CAD		MI					
Characteristic	OR (95% CI)	*p* value	OR (95% CI)	*p* value				
Smoking	2.53 (2.16–2.97)	<1.0E-25	3.89 (3.05–4.96)	<1.0E-25				
Diabetes*								
BMI (per 1 kg/m^2^)	1.08 (1.05–1.11)	1.11E-09	1.08 (1.04–1.11)	9.4E-07				
Glucose (per 1 mmol/L)	1.45 (1.36–1.55)	<1.0E-25	1.43 (1.31–1.56)	1.8E-15				
Triglycerides (per 1 mmol/L)	0.88 (0.83–0.94)	8.36E-05	0.87 (0.80–0.95)	0.001				
HDL-cholesterol (per 1 mmol/L)	0.20 (0.16–0.26)	<1.0E-25	0.17 (0.12–0.23)	<1.0E-25				
*PLA2G7* F vs V	0.80 (0.69–0.92)	0.002^a^	0.75 (0.62–0.90)	0.002^a^				
FF vs VV	0.66 (0.38–1.14)	0.05^b^	0.43 (0.19–0.96)	0.03^b^				
VF vs VV	0.72 (0.62–0.84)		0.78 (0.64–0.95)					

a p value for comparison of allelic frequency;

b p value for comparison of genotypic frequency. *no estimate in study 2 and meta-analysis, due to zero count in control group.

**Figure 1:**
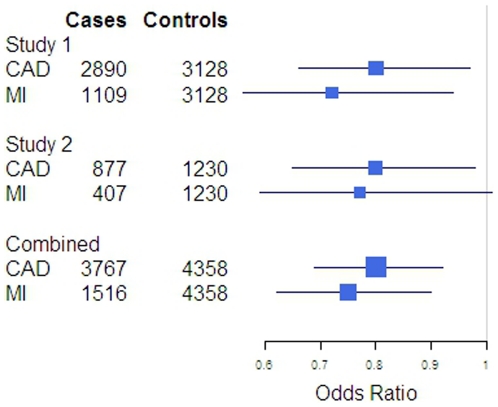
CAD and MI risk (OR and 95% CI) in carriers of the *PLA2G7* 279F compared to subjects homozygous for the common V279 allele are shown by study and combined using meta-analysis. An additive model was used therefore homozygous carriers of the 279F would be expected to have twice as much risk reduction as compared to heterozygous carriers. CAD – coronary artery disease and MI – myocardial infarction.

To consolidate these findings, the *PLA2G7* V279F variant was subsequently examined in Study 2, an independent dataset. The clinical characteristics of the participants in this study were very similar to the ones observed in Study 1 (Table 1), with the difference that a less restrictive age criteria was used. The prevalence of the *PLA2G7* 279F allele was almost identical in cases from Studies 1 and 2, and also in controls from these two independent studies; in particular, the prevalence of 279F in Study 2 was equally lower in cases with CAD (11.2%) and MI (11.3%) compared to controls (12.5%)(Table 2). In the logistic regression model, this allele tended to offer a protection against CAD with a magnitude and direction which were similar to the one observed in Study 1 [OR 0.80 (0.65–0.98), p = 0.04]. If an identical age criteria to the first study was used in Study 2, despite a smaller sample size the results were very similar [OR 0.81 (0.62–1.06), p = 0.13], therefore only results from the full sample are presented. Meta-analysis of the two studies revealed that the 279F null allele was associated with an overall 20% [OR 0.80 (0.69–0.92), p = 0.002] and 25% reduction [OR 0.75 (0.62–0.90), p = 0.002) in the risk of CAD or MI, respectively (Table 3 and Figure 1). The association with CAD was not solely dependent on the MI group, as an analysis of CAD without MI remained significant, OR 0.82 (0.73–0.98), p = 0.03.

One potential limitation of this study is the fact that not all cases and controls were collected from the same sites. In order to assess heterogeneity between sites, we examined the prevalence of the 279F allele in each site individually. The allele frequency was very consistent between sites, ranging from 11.3% to 11.8% for CAD cases and from 12.6% to 13% in controls (Table S1), without any overlap between the two groups.

## Discussion

In this study, the largest to date to explore the association between the *PLA2G7* V279F variant and CAD, we provide consistent evidence that this null allele offers a significant protection against CAD, in particular myocardial infarction, in Korean males.

The present data shows that carriage of one copy of the 279F null allele was associated with a 20% reduction in risk for CAD. Our finding is consistent with the expected reduction in CAD risk, when considered in light of prior reports showing that each allele is associated with a ∼50% reduction in plasma Lp-PLA2 activity [5]–[14]. In the meta-analysis by Thompson *et al* [1], a 50% reduction in Lp-PLA**_2_** activity/mass corresponded to ∼1.5–2.0 SD, depending on the studies included in the meta-analysis, and each SD was associated with an 11% reduction in risk of CAD. This is fully consistent with the estimate of 20% reduction obtained empirically here. The convergence and consistency of the present genetic results with the epidemiological data on Lp-PLA2 activity and CAD risk supports a causative role for Lp-PLA**_2_** in the generation of CAD.

Taken together, these findings support the use of pharmacological agents that would inhibit Lp-PLA**_2_** as a way to prevent CAD. Pharmacological inhibition of the enzyme using darapladib averages 80%, and therefore the effect may be more pronounced than the effect of heterozygous carriage of the null allele. While this offers promise for inhibition, it remains challenging to extrapolate the effect of a genetic defect, established since the time of conception, to a pharmacological intervention of limited duration, usually initiated during adult life. As an illustration, for a similar amplitude, reduction of LDL-cholesterol levels associated with a genetic variant in *PCSK9* appears to provide a stronger protection against CAD than would statins [26].

The present study has some limitations. First, we only report here data from men. The *PLA2G7* V279F and the 9p21 variants were originally typed in 1130 female cases (diagnosed before age 65) and 1680 controls (clinically devoid of CAD above age 55) from Study 1. No association was observed between V279F mutation and the risk of CAD in this dataset [OR 1.09 (0.85–1.39), p = 0.50]. However, for reasons which were unclear as the 9p21 locus has been equally associated with CAD in males and females, [21], [27] including in Asia [28], no association either was observed with this positive control [OR 1.11 (0.94–1.30)]. One possible explanation for these results is the presence of asymptomatic coronary artery disease among control females, and a corresponding misclassification leading to an absence of detectable association. In these conditions, and considering the fact that no women were available in Study 2 to increase power and replicate findings, it was felt more appropriate, and rigorous, to restrict the present report to males.

The present study is based on a cross-sectional, age-discordant case-control design. Because relatively younger CAD cases (who may have a higher genetic burden than older cases) were compared with older controls, one cannot exclude the possibility that the benefits of carrying the 279F allele may have been over-estimated here. This possibility, however, is unlikely, as similar ORs were observed in Study 2 which was not age-restricted. Another limitation of the study is that controls did not have any coronary angiogram performed on them, and some of them may have clinically asymptomatic CAD, which may have led to under-estimation of the effect of the variants tested here. Conversely, the angiographic case definition for CAD in non-MI individuals was relatively lenient, and there is a possibility of over-diagnosis of CAD. Still, the overall amplitude of the effect of the positive control 9p21 locus marker in the present study (a 28% increase in CAD risk, Table 3) matched quite closely the effect reported in other case-control studies performed in Korea [23], [24] and a recent meta-analysis on this marker [25] [OR 1.25 (1.21–1.29)], suggesting that the amplitude of the protective effect of *PLA2G7* 279F reported here is quite accurate. Next, in the present study, cases and controls were not all collected from the same centers. The genetic homogeneity of the South Korean population, [29] the fact that the allele frequencies did not differ markedly between recruitment centers and the fact that the significance of the results were not affected upon controlling for genomic control inflation factor measured in other studies (1.061) [29] are reassuring. If this inflation factor were applied to our results, the meta-p value marginally increased (0.0027 vs 0.002) and remained significant. Finally, Lp-PLA**_2_** levels in plasma were not measured in the present study. However, given the large, unambiguous body of published observations, [5]–[14] one can reliably anticipate that heterozygous carriage of 279F allele is associated with a ∼50% reduction in activity and homozygosity with almost complete absence of the enzyme in plasma.

In conclusion, the present study indicates that natural, genetic deficiency in Lp-PLA**_2_** activity due to carriage of *PLA2G7* 279F null allele offers a certain protection against CAD, in particular MI, in Korean men. As such, these results provide strong evidence for a causal relationship between Lp-PLA**_2_** and CAD, and additional support to the concept that pharmacological inhibition of Lp-PLA**_2_** represents an innovative way to prevent this disease.

## Methods

### Study Subjects

For Study 1, CAD cases were enrolled in the Seoul National University Hospital [30], Samsung Medical Center [24] and Yonsei Cardiovascular Hospital [31]. The diagnosis of CAD was based on the presence of MI or on angiographic findings. The diagnosis of MI was made using the World Health Organization criteria including symptoms, enzyme elevation and electrocardiographic changes. Coronary angiograms were interpreted by a consensus of two independent observers. CAD was defined as ≥50% luminal stenosis in one or more major coronary arteries. CAD cases were selected among CAD patients diagnosed up to age 60 years. Patients with the following conditions were excluded from participation: valvular heart disease, peripheral vascular disease, significant systemic disease, history of inflammatory disease and history of congestive heart failure with left ventricular ejection fraction <30%. Overall, Seoul National University Hospital, Samsung Medical Center and Yonsei Cardiovascular Hospital contributed *PLA2G7* V279F genotypes for 943, 526 and 1340 CAD cases, respectively [this genotype was missing for the remaining 81 cases (2.8%) due to technical reasons, see below] (Table S1).

Controls for Study 1 were healthy volunteers who participated in the Seoul National University Hospital genome study (n = 281 genotypes) [30], in the Cardiovascular Genome Study (n = 667) [24], in the Korean Health and Genome Study (KoGES, n = 1812) [29] or at the Health Promotion Center in University Hospitals (n = 317) [32], with missing genotypes for 51 individuals (1.6%). Controls were aged over 50 years and had not been diagnosed with any cardiovascular diseases at the time of recruitment. Coronary angiography was not performed on the controls since they had no evidence of CAD by symptoms, history or non-invasive testing (electrocardiography). Controls were studied using the same protocol in each center, including a standardized interview focusing on medical history, physical activity, medication, personal habits, a physical examination and blood testing.

Cases and controls from Study 2 were all recruited from the Cardiovascular Genomic Center at Yonsei University Medical Center [31] using a similar inclusion and exclusion criteria, but with a less restrictive age criteria (age <80 for cases and >40 years old for controls). A total of 877 CAD cases (407 MI) and 1230 non-CAD controls were included in Study 2. All cases and 1217 controls had complete genotype data. If the same age criteria from Study 1 are applied, there were 686 CAD cases and 701 non-CAD controls, with complete genotype information.

Written informed consent was obtained from each study participant, and the study protocol was approved by the ethics committee or institutional review board in each of the participating centers (Institutional Review Board of Human Research of Yonsei University, Institutional Review Board of Seoul National University Hospital, Samsung Medical Center Ethics Committee, National Institute of Health Ethics and the Institutional Review Board of the Korean Health and Genomic Study).

### Genotyping

Genomic DNA was prepared from peripheral blood samples using the Puregene DNA purification kit (Gentra, Minneapolis MN, USA). SNPs were genotyped using the TaqMan fluorogenic 5′ nuclease assay (ABI, Foster City CA, USA). The final volume of polymerase chain reaction (PCR) was 5 µl, containing 10 ng of genomic DNA and 2.5 µl TaqMan Universal PCR Master Mix, with 0.13 µl of 40X Assay Mix (Assay ID C__11915291_20 for *PLA2G7* RS16874954 and C__26505812_10 for 9p21 locus RS10757274). Thermal cycle conditions were as follows: 50°C for 2 min, 95°C for 10 min followed by 45 cycles of 95°C for 15 s and 60°C for 1 min. PCR was performed using a Dual 384-Well GeneAmp PCR System 9700 (ABI) and the endpoint fluorescent readings were performed on an ABI PRISM 7900 HT Sequence Detection System. Duplicate samples (n = 212 for V279F and n = 322 for 9p21) and negative controls were included to ensure genotyping accuracy. The genotyping error rate was 0.2%.

### Statistics

Hardy-Weinberg equilibrium was confirmed for both markers using standard approaches. For Studies 1 and 2, the clinical characteristics were compared between CAD patients and control subjects using the chi-square test for categorical variables and 2-tailed Student t-tests or Wilcoxon test (for non-normally distributed triglycerides levels) for continuous variables. The association between genotype and CAD was examined using logistic regression analysis adjusting for age, smoking status (former and current smoker *vs* never smoker), BMI, diabetes and plasma levels of glucose, triglycerides, HDL-cholesterol and LDL-cholesterol. As a large fraction of CAD cases were taking statins, LDL-cholesterol levels were corrected as recommended by Tobin [33] by multiplying LDL-cholesterol levels by a factor of 1.25, corresponding to a conservative average 20% reduction in LDL-cholesterol levels in subjects taking statins [34], [35]. Since the age distribution of cases (aged <60) and controls (aged >50) differed by design, age was included in the regression model, but the parameter estimate was not valid. To test for multi-collinearity between the genotype and covariates which could inflate any association, we calculated the variance inflation factor (VIF) and tolerance for all explanatory variables used in the linear regression analysis. All tolerances were close to.60 and none of the VIFs exceeded 2.2 in the regression model, providing no evidence of multi-collinearity. A meta-analysis of the two datasets was performed to provide an overall statistical evaluation of the effect of the V279F variant on CAD risk using the inverse variance weighted average method [36]. Statistical analyses were performed using SAS v9.0 (SAS Institute Inc, Raleigh NC, USA).

## Supporting Information

Table S1 **Genotype and allele frequencies of V279F (** ***PLA2G7*** **) and RS10757274 (9p21) by centers.**
(DOC)
