# Supplementary material for: Carriage of the V279F Null Allele within the Gene Encoding Lp-PLA2 Is Protective from Coronary Artery Disease in South Korean Males
Source: PLoS One. 2011 Apr 5;6(4):e18208. doi: 10.1371/journal.pone.0018208 (PMC3071750; doi:10.1371/journal.pone.0018208)
Supplement: Table S1 — Genotype and allele frequencies of V279F ( PLA2G7 ) and RS10757274 (9p21) by centers. (DOC) [file pone.0018208.s001.doc]

**Table S1: Genotype and allele frequencies of V279F (*PLA2G7*) and RS10757274 (9p21) by centers**

|  | **Study 1** | | | | | | | |  | **Study 2** | |
| --- | --- | --- | --- | --- | --- | --- | --- | --- | --- | --- | --- |
|  | **CAD** | | |  | **Control** | | | |  | **CAD** | **Control** |
|  | SNUH | Samsung Med. Centre | CGC Yonsei Univ. |  | SNUH | CGC Yonsei Univ. | KoGES | HPC Univ. Hospital |  | CGC Yonsei Univ. | |
| ***PLA2G7*** |  |  |  |  |  |  |  |  |  |  |  |
| VV | 744 | 412 | 1049 |  | 212 | 508 | 1389 | 236 |  | 692 | 933 |
| VF | 185 | 104 | 275 |  | 65 | 148 | 389 | 77 |  | 174 | 264 |
| FF | 14 | 10 | 16 |  | 4 | 11 | 34 | 4 |  | 11 | 20 |
| MAF* | 0.113 | 0.118 | 0.115 |  | 0.13 | 0.127 | 0.126 | 0.134 |  | 0.112 | 0.125 |
|  |  |  |  |  |  |  |  |  |  |  |  |
| **9p21** |  |  |  |  |  |  |  |  |  |  |  |
| AA | 236 | 111 | 346 |  | 93 | 188 | 574 | 124 |  |  |  |
| AG | 476 | 260 | 666 |  | 134 | 322 | 883 | 146 |  |  |  |
| GG | 221 | 152 | 325 |  | 56 | 123 | 359 | 48 |  |  |  |
| MAF | 0.492 | 0.48 | 0.492 |  | 0.435 | 0.449 | 0.441 | 0.381 |  |  |  |

*MAF: Minor Allele Frequency

SNUH: Seoul National University Hospital, Samsung: Samsung Medical Center, CGC Yonsei: Cardiovascular Genome Center of

Yonsei University Medical Center, KoGES: Korean Genome Epidemiology Study, HPC: the Health Promotion Center in University

Hospital
